# Auditory change detection in musically trained adolescents and young adults: An EEG–fMRI study

**DOI:** 10.1111/nyas.70008

**Published:** 2025-07-29

**Authors:** Vesa Putkinen, Katri Saarikivi, T. M. Vanessa Chan‐Devaere, Mari Tervaniemi

**Affiliations:** ^1^ Turku PET Centre, Turku University Hospital University of Turku Turku Finland; ^2^ Turku Institute for Advanced Studies University of Turku Turku Finland; ^3^ Cognitive Brain Research Unit, Department of Psychology, Faculty of Medicine University of Helsinki Helsinki Finland; ^4^ Department of Psychology University of Notre Dame Notre Dame Indiana USA; ^5^ Centre of Excellence in Music, Mind, Body, and Brain, Faculty of Educational Sciences University of Helsinki Helsinki Finland

**Keywords:** fMRI, mismatch negativity, musical training

## Abstract

Musical training has been associated with enhanced auditory processing, including superior preattentive sound discrimination. However, the neural mechanisms underlying these enhancements remain unclear. This study used electroencephalography (EEG) and functional magnetic resonance imaging (fMRI) to investigate auditory deviance detection in musically trained and untrained 16–20‐year‐old participants. They listened to a sequence of major chord standards interspersed with minor chord deviants while watching a movie without sound in two separate sessions, once during EEG recording and once during fMRI. As expected, musically trained participants exhibited larger mismatch negativity (MMN) and P3a amplitudes, indicating enhanced neural discrimination and attentional engagement with harmonic deviations. Surprisingly, fMRI revealed that the Control group showed greater activity in Heschl's gyrus for deviant versus standard chords. This indicates that the enhanced EEG responses in the Music group were accompanied by reduced hemodynamic activity in primary auditory areas. These findings highlight the value of multimodal approaches in studying neural differences between musically trained and untrained individuals and suggest that electrophysiological and hemodynamic measures capture distinct aspects of these differences.

## Graphical abstract

Electroencephalography (EEG) and functional magnetic resonance imaging (fMRI) were used to examine auditory deviance detection in musically trained and untrained 16–20‐year‐olds. Participants passively listened to a sequence of major chord standards and minor chord deviants during separate EEG and fMRI sessions. Musically trained individuals showed larger mismatch negativity and P3a responses, while untrained controls showed greater blood–oxygen level dependent (BOLD) activity in Heschl's gyrus.

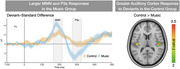

## INTRODUCTION

Musicianship is associated with enhanced auditory processing extending beyond conscious sound discrimination to preattentive change detection (for a review, see Ref.1). One of the most widely used measures of such auditory discrimination is the mismatch negativity (MMN), a negative component of the auditory event‐related potential (ERP) that reflects the brain's ability to detect changes in a sound stream by comparing incoming sounds to predictions based on previously heard sounds.^2^, ^3^ The MMN is typically elicited in an oddball paradigm, where a sequence of standard sounds is occasionally interrupted by a deviant sound that violates some regularity established by the standard sounds, whether in physical features or in higher order patterns. Importantly, the MMN is generated even in the absence of attention, indicating that it reflects an automatic auditory change detection process.

Studies have consistently demonstrated that musicians exhibit larger MMN responses than nonmusicians.^4^, ^5^ Longitudinal research further suggests that musical training in childhood is associated with increasing MMN amplitudes over time.^6^, ^7^ For example, we found that children undergoing musical training exhibited a greater increase in MMN amplitude with age for minor chord deviants within a sequence of major chord standards compared to their untrained peers, with no significant group differences at the onset of training.^7^ This indicates that musical training shapes neural sound discrimination as children develop and accumulate training.

The underlying neural mechanisms of the MMN have also been investigated with functional magnetic resonance imaging (fMRI). This research has implicated Heschl's gyrus and the superior temporal gyrus (STG) as primary generators of the MMN, with additional contributions from the right inferior frontal cortex, which may support higher order attention switching in response to sound changes.^8^, ^9^, ^10^, ^11^ For salient sound changes, the MMN is often followed by a positive response, the P3a, which is thought to reflect this attention‐shifting process.^12^ Electroencephalography (EEG)–fMRI studies have confirmed that activity in temporal regions scales with the magnitude of deviant sounds, though evidence for a deviance‐magnitude effect in frontal activation has been inconsistent.^13^

In the present study, we examined both ERP and fMRI measures of auditory deviance processing in musically trained and untrained participants aged 16–20 years to determine whether musical training is associated with differences in the neural mechanisms underlying automatic sound discrimination. The participants were from the same cohort as in our previous study,^7^ allowing us to investigate whether previously observed MMN differences persist into adolescence and early adulthood. The stimulus paradigm consisted of major chord standards along with minor chord deviants. In the fMRI experiment, we also included small deviants in which the third of the chord was slightly mistuned to examine whether group differences varied with deviance magnitude (cf. Koelsch et al.^5^).

Based on prior research, we expected larger MMN and P3a responses in musically trained adolescents, reflecting enhanced neural discrimination and attentional orienting to musical deviations. It remains unclear whether the advantage of musically trained individuals over untrained children in auditory discrimination persists into adolescence or diminishes over time, potentially due to the maturation of auditory processing abilities, as is seen in executive function test performance where the initially greater advantage for the music group decreases during this developmental stage.^14^ Additionally, the use of fMRI allowed us to explore whether these neural differences would be reflected in the activity of auditory processing regions such as Heschl's gyrus, the STG, and inferior frontal cortex.^13^ By integrating EEG and fMRI, this study aimed to provide a more comprehensive understanding of how musical training influences neural responses to auditory changes and the hemodynamic activity associated with enhanced electrophysiological responses.

## METHODS

### Participants

Forty‐two adolescents participated in the study. Four participants were excluded from the fMRI analysis due to excessive head movement. The Music group consisted of adolescents who began playing a musical instrument approximately at the age of 7 (*N* = 19, age range 15.8–20.7, mean age 18.1 years, six males). They had attended a public elementary school that included solo instrument lessons, music theory, and choir and orchestra ensembles as part of its regular curriculum. The Control group consisted of adolescents without formal music training or musical hobbies, who attended or had attended a standard elementary school (*N* = 19, age range 16.2–20.7, mean age 18.4 years, nine males). A subset of these participants (Music group: *N* = 18, mean age 18.0, five males; Control group: *N* = 16, mean age 18.3, seven males) also took part in an ERP experiment employing the chord MMN paradigm used in our previous study.^7^ For participants who took part in both the fMRI and EEG experiments, data were collected on separate days with the order of sessions counterbalanced across participants. Participants were compensated for their time with movie vouchers. Prior to the start of the first session, written informed consent was obtained from all participants; for those under 18, consent was also obtained from a parent or guardian. The experimental protocol was approved by the Ethical Committees of the former Department of Psychology and of the Faculty of Behavioural Sciences, both at the University of Helsinki, Finland.

### ERP paradigm

In the chord paradigm, standard stimuli (*p* ≈ 0.84, *N* = 455) consisted of the C‐major triad chords, while deviant stimuli (*p* ≈ 0.16, *N* = 75) were C‐minor triad chords with identical acoustic features as in the fMRI experiment (see above). The stimuli were presented with a stimulus‐onset asynchrony (SOA) of 725 ms. The total sequence duration was approximately 6.5 min. The participants watched a movie without sound during the experiment. To maintain consistency with our earlier ERP study on the same participant cohort,^7^ the ERP paradigm used here employed a slower SOA than the fMRI paradigm and did not include the small deviant stimuli that were introduced in the latter paradigm (described below).

### fMRI paradigm

The stimuli were presented using an oddball paradigm consisting of frequent standard chords and infrequent deviant chords. All stimuli were composed of three sine‐wave tones and had a duration of 125 ms. The standard stimulus was a C‐major chord (fundamental frequencies: 262, 330, and 392 Hz).

The paradigm included two types of deviant stimuli: a small deviant, in which the frequency of the third (i.e., the intermediate pitch) was lowered by 50% of a semitone, and a large deviant, in which the third was lowered by a full semitone, corresponding to a C‐minor chord.

Stimuli were delivered through MRI‐compatible earphones throughout the scanning session while participants watched a muted nature documentary. The paradigm was adapted from Schönwiesner et al.^13^ The stimuli consisted mostly of standards, while deviant stimuli appeared infrequently at 2, 4, 5, 6, or 7 s before the following repetition time (TR). Across the three runs, the number of trials with these deviant presentation timings was 18, 42, 42, 42, and 30, respectively. Additionally, 30 trials without deviants were included as a baseline measure. The total duration of this task was 32 min.

Unlike the ERP paradigm, the fMRI paradigm included a small deviant to increase task difficulty and sensitivity to detect potential group differences in late adolescence. This also allowed us to explore effects of deviant magnitude in line with the paradigm used by Schönwiesner et al.^13^

### EEG acquisition and analysis

EEG data were continuously recorded using a BioSemi Active‐Two system (BioSemi) with a sampling rate of 512 Hz. The recordings were obtained from 64 active Ag–Cl electrodes, arranged according to the International 10–20 system, along with additional electrodes placed at the nose and on the left and right mastoids. To monitor ocular activity, electrooculography (EOG) was recorded using two electrodes: one positioned below the left eye and the other at the lateral aspect of the left outer canthus.

Preprocessing and analysis of the EEG data were carried out in MATLAB with the EEGLAB toolbox.^15^ First, a high‐pass filter at 0.5 Hz (Hamming windowed sinc FIR filter) was applied to the continuous data. The data were then segmented into epochs spanning from 100 ms before to 500 ms after stimulus presentation and were re‐referenced to the average signal from the two mastoid electrodes. To address artifacts, independent component analysis (ICA) was performed after an initial step of visually inspecting and removing noisy epochs, as well as identifying and excluding bad channels. The resulting independent component (IC) topographies were examined to detect and eliminate components associated with ocular and motion artifacts. Following ICA, a low‐pass filter at 30 Hz was applied. Any remaining epochs with voltage fluctuations exceeding ±100 µV were automatically discarded. Subsequently, any removed channels were interpolated, and the epochs were averaged separately for standard and deviant sound conditions. Mean amplitude differences (deviant‐minus‐standard) were computed over 50‐ms time windows centered at 190 ms (MMN) and 300 ms (P3a) after stimulus onset. These time windows were selected to optimally capture the responses of both groups at electrode Fz, where the amplitudes were largest. This selection provided a good compromise between individual variability in peak latencies and the need for a consistent measure across the groups.

To analyze the MMN and P3a amplitudes, we conducted a mixed‐design analysis of variance (ANOVA) using the *ezANOVA* package^16^ in R for the response amplitudes at F3, Fz, and F4. These electrodes were selected based on the frontocentral distribution of the MMN and P3a responses. The independent variables included the within‐subjects factor of Laterality (Left/F3, Midline/Fz, Right/F4) and the between‐subject factors of Group (Music, Control) and Age. The ANOVA was performed using Type III sums of squares to accommodate unbalanced designs if present.

### fMRI acquisition and analysis

Scanning was conducted with a Siemens 3‐Tesla magnet at the Advanced Magnetic Imaging Centre of Aalto University in Espoo, Finland. A high‐resolution structural image was acquired from each participant using a T1‐weighted sequence (3D MP‐RAGE) with a resolution of 1 mm^3^, which was subsequently used to set the functional images to cover bilateral temporal and inferior frontal cortices. Blood–oxygen level dependent (BOLD) contrast images were acquired using gradient echo planar imaging (TR/TE 9000 ms/32 ms) with a head quadrature receiver/transmitter coil. Functional images consisted of 20 ascending slices with in‐plane resolution of 3 × 3 mm and slice thickness of 3 mm. Slices were acquired in direct temporal succession in the first 1200 ms of each TR followed by 7800 ms of stimulus presentation without scanner noise. This sparse sampling paradigm reduces the effect of scanner noise on the participant's response to an auditory stimulus.

The fMRI data preprocessing was performed using SPM12 (Wellcome Centre for Human Neuroimaging) implemented in MATLAB (MathWorks Inc.). To correct for head motion, all functional images across the three runs were realigned to the mean functional image using a six‐parameter rigid body transformation. The estimated motion parameters were stored for subsequent use as nuisance regressors in the first‐level general linear model (GLM) analysis.

The mean functional image was then coregistered to the participant's structural T1‐weighted image. The structural T1 image was segmented into gray matter, white matter, and cerebrospinal fluid using SPM's unified segmentation approach. The deformation fields generated during this step were used to normalize the functional images to Montreal Neurological Institute (MNI) space. Finally, the normalized functional images were spatially smoothed using a Gaussian kernel with a full‐width at half‐maximum (FWHM) of 8 mm to improve the signal‐to‐noise ratio and accommodate intersubject variability in functional anatomy.

First‐level GLM analyses were conducted separately for each participant to estimate individual subject responses to the trial types. High‐pass filtering was set to 128 s to remove low‐frequency signal drifts. To account for head motion, six motion parameters were included as nuisance regressors. The design matrix included regressors for standard, small deviant, and large deviant trials along with the motion parameters. Subject‐wise images for the contrast of interest were used in second‐level group analyses.

A region‐of‐interest (ROI) was conducted by extracting the mean beta values for small and large deviants and standard in left and right Heschl's gyrus where activity have been consistently reported in previous fMRI studies on MMN generators. The ROI binary maps were generated using the Harvard–Oxford structural atlas.

The mean beta values were analyzed with a 2 × 3 × 2 repeated measures ANOVA with group (Music, Control) as a between‐subjects factor and Contrast (Large Deviants, Small Deviants, Standards), Hemisphere (Left, Right) as within‐subjects factors. Greenhouse–Geisser correction was used if the sphericity assumption was violated. Interactions of interest were further explored using reduced follow‐up ANOVAs conducted on subsets of the data (see the Results section).

## RESULTS

### ERP

**FIGURE 1:**
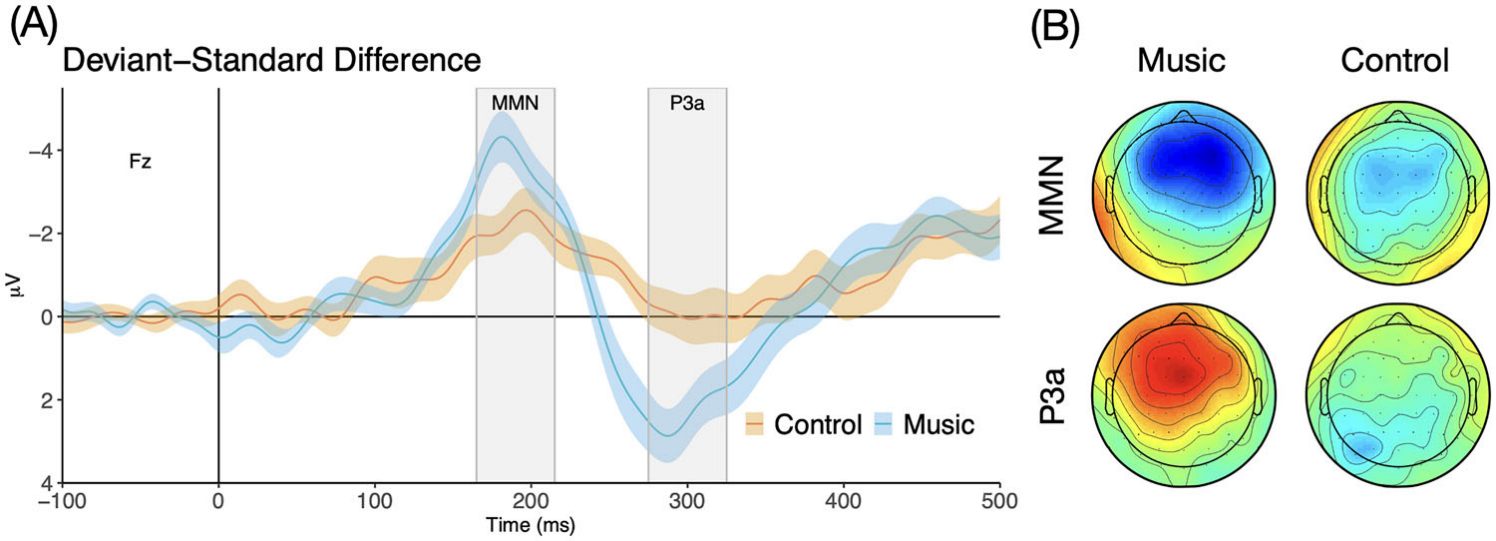
(A) Deviant − standard difference responses for the Music and Control groups at Fz. The shaded bars represent the time windows used to compute the participant‐wise MMN and P3a mean amplitudes. The shaded regions around the responses represent the standard error of the mean. (B) The distribution of responses across all electrodes within the MMN and P3a time windows for the Music and Control groups. Color indicates the polarity of the response (blue = negative, red = positive), and color intensity reflects the response amplitude on an arbitrary scale.

Figure 1 illustrates the deviant − standard difference responses. Compared to the Control group, the Music group showed larger MMN (main effect of Group: *F*(1, 32) = 4.450, *p* < 0.05, *η* ^2^ = 0.112) and P3a than the Control group (main effect of Group: *F*(1, 32) = 8.055, *p* < 0.01, *η* ^2^ = 0.190). Follow‐up *t*‐tests at Fz revealed no significant group differences in standard response amplitudes in either the MMN or P3a time windows (*p*s > 0.22), whereas deviant response amplitudes differed significantly between groups (MMN: *t*(32) = −2.1, *p* < 0.05; P3a: *t*(32) = 2.1, *p* < 0.05), supporting the interpretation that the observed group differences reflect responses to the deviants rather than to the standards. No other significant main effects or interactions were observed.

### fMRI

**FIGURE 2:**
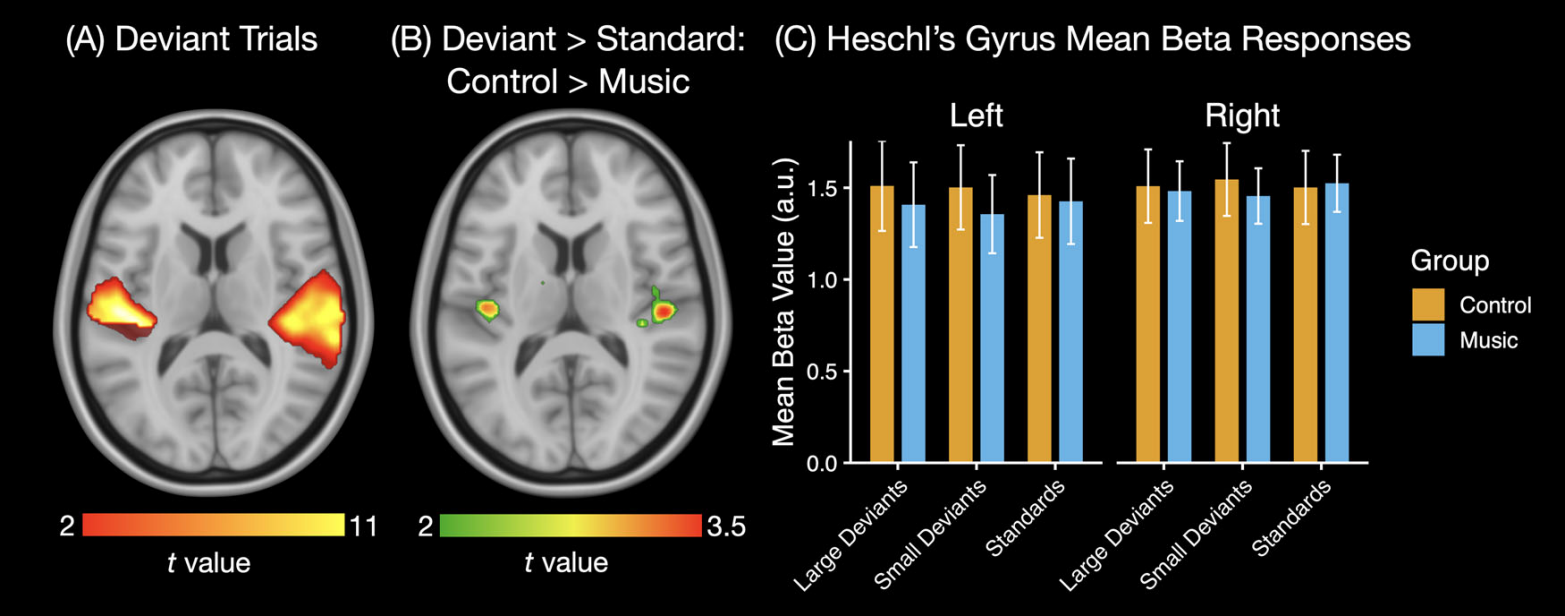
(A) Brain regions exhibiting increased BOLD response during all deviant trials (i.e., aggregated over large and small deviants), with data thresholded at *p* < 0.001 and FWE corrected at the cluster level. (B) Regions where the Control group showed a greater response to large and small deviants > standard contrast compared to the Music group. (C) Mean beta values per trial type, group, and hemisphere. The data in (B) are presented to aid in the interpretation of the ROI analysis results and are thresholded at *p* < 0.05, uncorrected. BOLD, blood–oxygen level dependent; FWE, family‐wise error; ROI, region‐of‐interest.

The deviant trials elicited bilateral activation in the auditory cortices (Figure 2A). In the whole‐brain voxel‐wise analysis, neither the contrast between large and small deviants nor the differences between the Music and Control groups remained significant after cluster correction. However, the ROI analysis revealed that the Control group exhibited a larger deviant > standard difference in Heschl's gyrus compared to the Music group (Group × Contrast interaction: *F*(2, 62) = 3.545, *p* < 0.05, *η* ^2^ = 0.035). This result was further supported by voxel‐wise comparison of the groups for the all deviant trials > standard trials contrast, although this analysis did not survive the cluster correction and was significant only at the voxel level (Figure 2B,C). No other significant effects were found in the ROI analysis.

To further investigate the significant Group × Contrast interaction observed in the full ANOVA, we first conducted an ANOVA on deviant‐minus‐standard difference responses with Group (Music, Control) as a between‐subjects factor, and Deviant Magnitude (Small, Large) and Hemisphere (Left, Right) as within‐subjects factors. This analysis revealed a significant main effect of Group (*F*(1, 31) = 4.55, *p* < 0.05, *η* ^2^ = 0.082), but no interaction with Deviant Magnitude, indicating that the group difference in deviant‐related BOLD responses did not significantly vary by deviant size. To assess potential group differences in standard responses, we then conducted a separate ANOVA including only the standard trials. This analysis revealed no significant main effect of Group suggesting that the interaction observed in the full model was not driven by group differences in responses to the deviant stimuli.

## DISCUSSION

We examined electrophysiological and hemodynamic brain responses to minor chord deviants within a sequence of major chord standards in musically trained and untrained adolescents using ERPs and BOLD–fMRI. The ERP results revealed larger MMN and P3a responses in the Music group compared to the Control group, suggesting enhanced neural discrimination of the deviants and standards in musically trained adolescents. In contrast, the fMRI data showed greater deviant > standard responses in the auditory cortex for the Control group, indicating less engagement of primary auditory regions for deviant detection in the Music group. This pattern suggests that enhanced automatic auditory change detection in musically trained individuals is accompanied by lower hemodynamic activity in the primary auditory cortex, possibly reflecting more efficient processing of musical sound changes in this region.

### Enhanced MMN and P3a in musically trained adolescents

The enhanced MMN response demonstrates improved neural discrimination of major and minor chords in musically trained adolescents, replicating our previous findings from the same participants when they were 9–13 years old.^7^ In our earlier study, no significant differences were observed between the Music and Control groups, suggesting that the enhanced MMN in musically trained adolescents emerged as a result of training rather than pre‐existing differences, supporting the idea that musical training shapes auditory processing abilities over time.

Since distinguishing between minor and major chords is fundamental in Western tonal music, we expected that the Control group might have developed comparable neural discrimination abilities with age and increased exposure to music. For instance, one study reported no significant differences in MMN or P3a responses to major versus minor chords between adult musicians and nonmusicians.^17^ Their results, along with other research, indicate that adult nonmusicians can develop sophisticated, implicit music perception abilities through lifelong informal exposure to their culture's music.^18^ However, our findings suggest that the advantage in neural sound discrimination for these stimuli persists into adolescence or early adulthood, rather than diminishing with increasing maturation and musical exposure among untrained participants.

It is worth noting that the difference between the major chord standards and minor chord deviants is ultimately a physical pitch difference, with the third of the chord being one semitone lower in minor chords than in major chords. Therefore, our paradigm cannot determine whether participants relied solely on this physical distinction or whether their discrimination was based on more abstract, categorical representations of major versus minor chords. However, a prior study conducted partly with the same participants, controlled for low‐level frequency differences by using various inversions of major chord standards and minor chord deviants and found that children can extract abstract major and minor categories from auditory stimuli, with musically trained children exhibiting enhanced neural sensitivity to them.^19^

As in our previous study,^7^ the Music group also exhibited an enhanced P3a response. The P3a is commonly interpreted as an index of attentional switching,^12^ suggesting that changes from major to minor chords were more attention‐catching for musically trained children compared to their untrained peers. P3a enhancement has also been observed in adult musicians.^20^, ^21^, ^22^ However, in a separate study with the same participants where novel sounds were embedded in a sequence of standard tones, and participants performed a concurrent visual task, the P3a response to novel sounds was reduced for the musically trained.^23^ This suggests that musical training can both enhance or diminish P3a responses depending on the nature of the stimulus and the demands of the concurrent task. Musicians may exhibit heightened sensitivity to subtle musical changes, resulting in a stronger P3a for musical deviations, while also demonstrating a refined ability to suppress irrelevant auditory distractions leading to a reduced P3a for novel sounds. This pattern suggests an association between musical training and enhanced attentional control in a context‐dependent manner, with improved sensitivity to meaningful auditory cues and a stronger ability to filter out extraneous information.

### Reduced Heschl's gyrus activity in musically trained adolescents

The finding that the Control group exhibited a larger Heschl's gyrus response than the Music group for the deviant > standard contrast—indicating lower hemodynamic activity in the auditory cortex for musically trained adolescents—is somewhat surprising. One might expect expertise to be associated with enhanced neural responses in task‐relevant regions, yet in this case, musical training appears to be linked to a decreased BOLD signal in primary auditory areas.

One possible explanation is that musically trained adolescents recruit these regions more efficiently for deviant detection, requiring less neural activation to achieve the same perceptual outcome. This could reflect a refinement in auditory processing where trained individuals rely on more specialized and automatic neural mechanisms developed through years of musical experience. The notion that expertise leads to more efficient rather than greater neural activation is supported by findings from other domains where experts often show reduced activation in task‐relevant regions.^24^ Interestingly, our previous fMRI study with the same participants found that the Music group also exhibited lower BOLD responses during an executive functions task.^14^ This parallel suggests that the reduced hemodynamic activity in musically trained adolescents may not be limited to auditory processing but could extend to broader neural efficiency across cognitive domains. Musical training has been associated with enhancements in executive functions, and it is possible that a more streamlined neural response underlies both cognitive control and auditory discrimination. However, the reduced BOLD response in musically trained participants alone does not allow us to determine what this efficiency represents—whether, for example, it reflects differences in cognitive strategy, neural computations, metabolic demands, or network organization.^25^

There were also differences between the ERP and fMRI paradigms, such as the SOA and the inclusion of small deviants in the fMRI paradigm, which were implemented to optimize stimulus presentation for each research modality and ensure consistency with previous studies. While we cannot rule out the possibility that these differences influenced our results, they are unlikely to fully account for the enhanced ERP responses and reduced BOLD responses observed in the Music group. However, it is important to note that the fMRI data may not have been sensitive enough to capture the group effects, as group differences only emerged in the ROI analysis. Furthermore, the fMRI data did not show the expected effects of deviance magnitude or the frontal activation typically associated with MMN and P3a responses. Notably, the large effect size observed in the ERPs for the P3a group difference suggests that the absence of frontal activity is unlikely to be solely due to a lack of statistical power: Cohen's *d* for the P3a amplitude difference between groups at Fz was 0.96, meaning that if a comparable effect size were present in the BOLD response, it would likely have been detected. These considerations raise the possibility that the brief neural activity underlying the MMN and P3a group differences may not be sufficient to produce a detectable hemodynamic response in fMRI. The divergence between ERP and fMRI findings—where musically trained individuals exhibited enhanced MMN and P3a responses but no corresponding increase in BOLD activity—suggests that early, transient neural activity related to group differences in the detection of subtle musical sound changes does not necessarily translate into sustained hemodynamic responses measurable with fMRI.

## CONCLUSION

Our findings indicate that enhanced neural discrimination of musical deviant sounds in musically trained children persists into adolescence, suggesting long‐lasting effects of musical training on auditory processing. While musically trained adolescents exhibited larger MMN and P3a responses, their lower BOLD activity in Heschl's gyrus may reflect more efficient recruitment of primary auditory regions for deviant detection. The complementary insights provided by ERP and fMRI emphasize the importance of multimodal approaches in understanding how musical training shapes auditory processing.

## AUTHOR CONTRIBUTIONS

Vesa Putkinen, Katri Saarikivi, and Mari Tervaniemi designed the study. Vesa Putkinen, Katri Saarikivi, T. M. Vanessa Chan‐Devaere, and Mari Tervaniemi wrote the paper. Vesa Putkinen, Katri Saarikivi, and T. M. Vanessa Chan‐Devaere collected and analyzed the data.

## CONFLICT OF INTEREST STATEMENT

The authors declare no conflicts of interest.

## Data Availability

The data that support the findings of this study are available upon request from the corresponding author. The data are not publicly available due to privacy or ethical restrictions.
